# Analysis of Stability and Variability in Sensor Readings from a Vehicle Weigh-in-Motion Station

**DOI:** 10.3390/s24248178

**Published:** 2024-12-21

**Authors:** Artur Ryguła, Krzysztof Brzozowski, Marcin Grygierek, Agnieszka Socha

**Affiliations:** 1 Department of Transport, Faculty of Management and Transport, University of Bielsko-Biala, Willowa 2, 43-300 Bielsko-Biała, Poland; kbrzozowski@ubb.edu.pl; 2 Department of Geotechnics and Roads, Faculty of Civil Engineering, Silesian University of Technology, Akademicka 5, 44-100 Gliwice, Poland; marcin.grygierek@polsl.pl; 3 APM PRO, Chochołowska 28, 43-346 Bielsko-Biała, Poland; agnieszka.socha@apm.pl

**Keywords:** weigh-in-motion, strain-gauge sensors, stability, load spectra, steering axle

## Abstract

This study presents a detailed analysis of the stability of weigh-in-motion sensors used at vehicle weighing stations. The objective of this research was a long-term assessment of reading variability, with a particular focus on the sensors’ application in automated measurement stations. These investigations constitute a critical component of modern traffic management systems and vehicle overload control. The analysis covered the period from 2022 to 2024, incorporating data from vehicles participating in regular traffic as well as dedicated control runs using vehicles with known wheel and axle load distributions. The study also considered changes in road surface conditions, particularly rut depth, and their variations over the examined period. The findings revealed that, despite the lack of station calibration over the three-year period, the observed parameters exhibited only minor changes. These results confirm the high stability of the applied measurement system and its ability to maintain measurement accuracy over extended operational periods, which is essential for its practical application in real-world traffic conditions.

## 1. Introduction

Nowadays, road transport is extensively monitored through automated systems utilising networks of sensors embedded in the roadway, systems based on computer vision, and systems using hybrid approaches. For freight transport, the core monitoring network consists of weigh-in-motion (WIM) stations, which are responsible for measuring axle loads, vehicle weights, and dimensions. These stations are equipped with sensors that detect the contact force exerted by a vehicle’s wheels as the vehicle passes through the station [1]. Accurate interpretation of the signals requires correct vehicle classification, which is achieved using specialised algorithms to determine axle spacings, supplemented by verification from video camera recordings. The station’s equipment allows for simultaneous monitoring of fundamental parameters of freight vehicle traffic flows, such as traffic volume, vehicle speeds, and classifications based on permissible gross weight and axle configurations. Monitoring of vehicle weight and parameters is also provided by systems developed for the evaluation of site-specific traffic loads on road bridges and for structure health monitoring, such as bridge weigh-in-motion (B-WIM) systems, as well as systems based on merging collected measurement data and simulation methods for traffic loads on road bridges [2,3]. Data gathered by weigh-in-motion stations are applied in studies of freight transport characteristics [4,5,6], pavement durability [7,8,9], and assessments of traffic safety and environmental impact [10,11,12]. Therefore, maintaining high data accuracy is critically dependent on managing the measurement error levels.

The total error of a single measurement within the WIM system combines systematic error from the method used to determine axle loads and random error. Both types of error are influenced by external factors and limitations of the sensors themselves [13]. Systematic error is typically corrected through proper system calibration, which involves comparing measurements against static axle loads of control vehicles in accordance with proper standards [14,15]. An example of such calibration was presented in [16], which proposed replacing calibration coefficients with calibration functions. However, WIM systems tend to exhibit drift and an increase in systematic error over time. The causes and rates of this phenomenon vary, influenced by factors such as the deteriorating quality of the pavement in which the sensors are embedded (pavement smoothness and geometry) and, consequently, the precision of the sensor response to wheel contact. Studies [17,18] have also shown that systematic error can fluctuate due to temperature variations and changes in vehicle traffic speed. A potential solution to this issue was presented in [19], proposing an algorithm for continuous calibration of WIM systems.

The second factor affecting measurement reliability, random error, may result from various factors related to the vehicle, sensor properties, and environmental conditions [20,21,22]. Controlling these factors is critical for ensuring measurement reliability, particularly in the context of enhancing enforcement of axle load and gross vehicle weight (GVW) limits. In direct enforcement systems, high precision and evaluation of factors affecting the measurements are essential [23,24,25].

Consequently, the primary challenge in maintaining the accuracy of these systems is the continuous monitoring of measurement reliability. This issue has been addressed in the literature, where certain indicators have been proposed to monitor changes in systematic error values. One such indicator is the steering axle load of a reference vehicle, used, for example, in [15] to define the standard steering axle load spectra. In [26], the steering axle load of a reference vehicle was employed to formulate a self-calibration method for the system. Another quality indicator for WIM station measurement data is the gross vehicle weight distribution for a reference vehicle [27,28], as well as tandem axle normalised axle load spectra, which typically exhibit a bimodal distribution due to the presence of loaded and unloaded trucks.

Recently, a method for estimating the systematic errors in the WIM system was proposed in [29]. The accuracy of the WIM system equipped with bending plates and quartz piezo load sensors was analysed in relation to axle load spectra attributes for single axles and tandem axles. It was found that the single-axle bias can be accurately estimated using differences in single-axle load means. On the other hand, the tandem-axle bias can be estimated using changes in the mean value for the loaded vehicle of the reference category. Moreover, the data show that the tandem-axle biases are significant contributors to gross vehicle weight errors. In another recently published work [30], WIM data consistency based on temporal axle load spectra was analysed. The results obtained led to the recommendation of calibration frequencies of at least every year for WIM stations with quartz piezo sensors and more frequent than yearly for piezo cable sensors. However, for WIM stations equipped with bending plate sensors, the results show that calibration frequencies greater than yearly may be acceptable. In summarising the results presented in both cited works, the changes in GVW and axle load spectrum parameters have a high correlation with the WIM measurement bias and, thus, can be used to identify calibration needs.

This study focuses on monitoring the stability of a WIM system [31] by analysing signal patterns recorded by weigh-in-motion sensors installed on a national road in Poland during the period from 2022 to 2024. The station was equipped with sensors and algorithms designed to monitor factors influencing random error levels. Specifically, the study analysed signal patterns recorded for individual wheels on each axle by strain-gauge sensors embedded in the road surface. The pavement quality was continuously monitored during the analysed period. Thus, this study contributes to the understanding of sensor drift phenomena in relation to measurement accuracy, as well as the impact of systematic error changes on steering axle load spectra and gross vehicle weight spectra, which are used as indicators of station stability.

## 2. Materials and Methods

### 2.1. WIM Station

**Figure 1:**
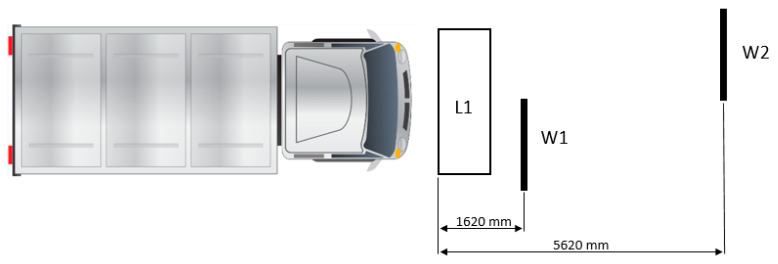
The layout of the WIM station. The size of sensors W1 and W2 is 1.75 × 0.07 m, with a sensitivity of 0.825 mV/V at 3000 kg [32]. Loop L1’s dimensions are 2.8 × 1 m.

The WIM station, which is the subject of the analysis, is located in Poland on the DK44 road in Mikołów-Śmiłowice. A detailed description of the station is provided in [21]. The devices constituting the station were installed in 2020 as part of the research and development project titled ‘Intelligent weight-in-motion system’ financed under the Regional Operational Programme for Śląskie Voivodeship for the years 2014–2020. The calibration of the station was conducted in 2021. During the period from 2022 to 2024, no calibration or maintenance operations were performed on the WIM sensors. For the purpose of the analysis, a basic sensor layout was selected, which includes an induction loop (L1) and two strain-gauge load sensors (W1, W2) (Figure 1). This arrangement ensures compliance with class B(+7) according to the COST 323 specification [15].

### 2.2. Pavement

**Figure 2:**
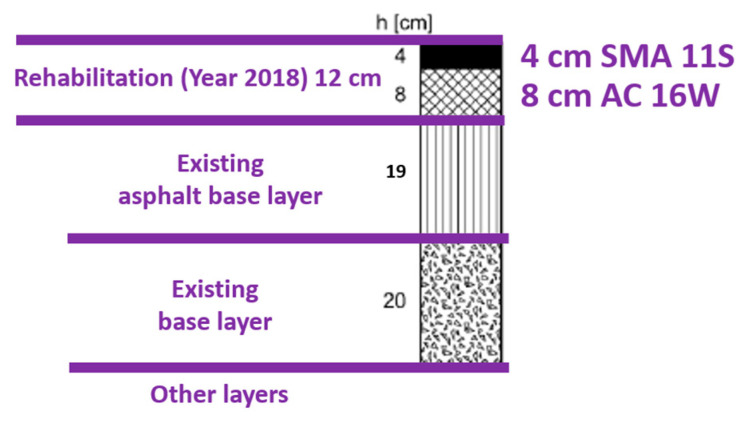
The road pavement construction at the WIM station.

An important element of the WIM station is the type of road surface, which plays a key role in the accuracy and stability of the measurement process. The road section under consideration features a flexible pavement consisting of the following layers: 31 cm of asphalt mixtures, a 20 cm base layer (unbound aggregate), and an improved subgrade. In 2018, the upper mineral-asphalt layers (wearing course and binder course) were replaced (Figure 2).

Between 2018 and 2021, measurements were conducted on both the geometric characteristics of the pavement and its deflections to assess load-bearing capacity. From 2022 to 2024, the focus shifted to the measurement of rut depths. Pavement deflections were measured using a dynamic deflectometer (FWD—Falling Weight Deflectometer), while rut depths were measured using the wedge and straightedge method.

### 2.3. Road Traffic Characteristics

During the analysed period, from the beginning of 2022 to June 2024, a total of 5.4 million vehicles passed through the WIM station, of which 650,000 were vehicles with a gross vehicle weight greater than 3500 kg. The daily traffic volumes at the WIM station in each year, along with the percentage share of vehicles with GVW > 3500 kg in the traffic, are presented in Table 1.

**Figure 3:**
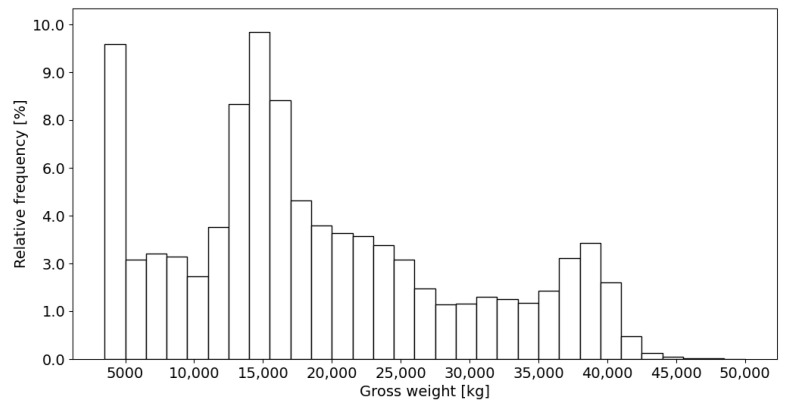
The distribution of the gross vehicle weight of the vehicles with GVW > 3500 kg in the period 2022–2024.

The data presented in Table 1 indicate that during the analysed period, a decrease in the average vehicle traffic flow was observed, including a reduction in the share of vehicles with GVW > 3500 kg. Overall, the change in the average daily traffic volume during this period amounts to 16%, accompanied by a proportional decrease in the share of vehicles weighing more than 3500 kg. The distribution of the gross vehicle weight recorded while passing through the station is shown in Figure 3.

Analysis of the vehicle weight distribution histogram indicates a significant proportion of vehicles with a gross vehicle weight up to 5000 kg, which represents the second-largest category in traffic flow. In contrast, the share of the heaviest vehicles, with a GVW of 40,000 kg, is relatively limited. Additionally, the contribution of overloaded vehicles within the total traffic volume of vehicles with a GVW greater than 3500 kg is negligible. During the observed traffic flow, nearly 340,000 vehicles classified as category 5 according to COST 323 (tractor with semi-trailer supported by tridem axles) were recorded. The weight of vehicles in this category ranges from 12,000 kg to over 40,000 kg for overloaded vehicles. The results obtained from the passage of vehicles in this category form the basis for the analyses presented in the subsequent sections of the study.

### 2.4. Control Runs

**Figure 4:**
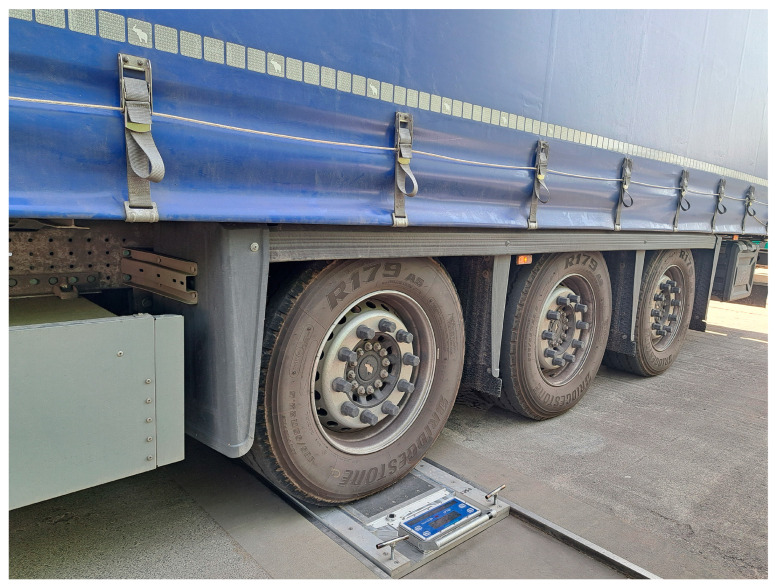
Static wheel load assessment using Intercomp LP788 scales.

The reference point for the conducted analyses is the results of the verification of the systematic error in dynamic weighing, collected as part of the control studies. For this purpose, in April 2023 and May 2024, a series of control runs were conducted through the WIM station using three different trucks (2-axle, 3-axle, and 5-axle) with known gross vehicle weight and axle/wheel load distributions. These values were verified using static weigh-in-motion scales Intercomp LP788 (Figure 4) with an error of 0.5%. Every vehicle completed 13 runs in 2023 and 13 runs in 2024.

## 3. Results and Discussion

The assessment of the stability and variability of axle load sensor readings requires an evaluation of conditions in relation to the pavement state, a comparison of histograms of gross vehicle weight distribution across different years, and the derived probability density functions of vehicle weight for the considered reference category, steering axle load spectra, and wheel load spectra for the steering axle.

### 3.1. Pavement State Analysis

**Figure 5:**
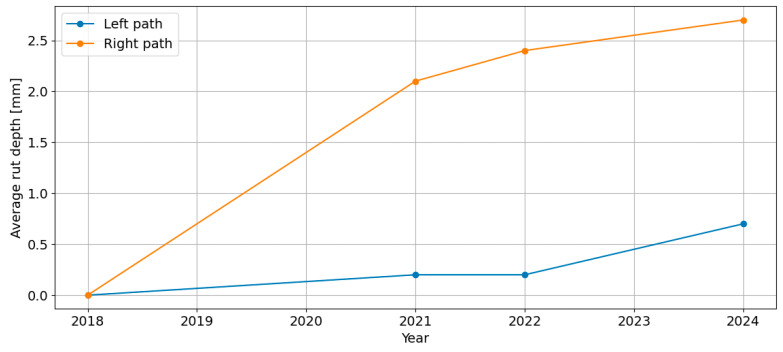
The increasing average rut depth for the entire section, with a breakdown by wheel path, from 2018 to 2024.

The results of the FWD measurements are described in [21]. These measurements classified the pavement as site I (Excellent) according to COST 323 [15] for so-called “flexible pavements”. In the analysis of rut depth, there is a difference between the left and right wheel paths. The rut in the right wheel path is deeper than in the left one. In analysing the average rut depth separately for the left and right wheel paths, it is noticeable that there was a significant increase in depth in the right wheel path before the analysed period, i.e., before 2022. In the subsequent years, the increase in rut depth in both paths is similar (Figure 5).

**Figure 6:**
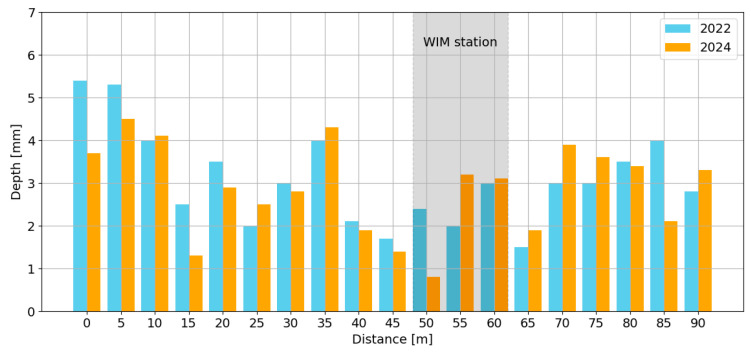
The distribution of the rut depth difference between the right and left wheel paths in the years 2022 and 2024.

**Figure 7:**
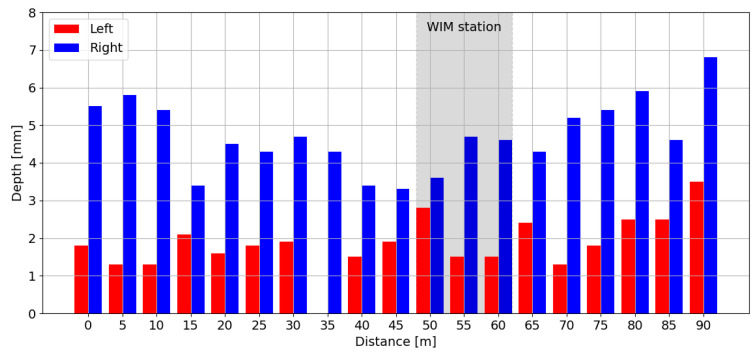
The distribution of depth in the left and right wheel paths according to measurements in 2024.

During the analysed period, the difference between the rut depth in the right and left wheel path over a 100 m section ranged from 0.8 mm to a maximum of 5.4 mm (Figure 6). The distribution of rut depths in 2024 is shown in Figure 7, which indicates that the maximum rut depth in the right wheel path no longer meets the requirements for pavements classified as site I (Excellent) according to COST 323 (rut depth ≤ 4 mm). From 2024 onward, the pavement meets the requirements for site II (Good) (rut depth ≤ 7 mm).

### 3.2. Weight Distributions for the Reference Vehicle Category

**Figure 8:**
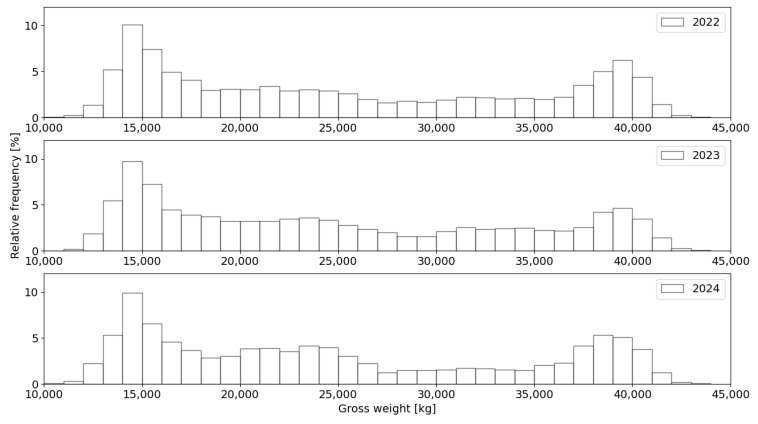
Total weight distribution for reference vehicle category.

The histograms of the total weight distribution for the reference vehicle category in the individual years of the analysed period are shown in Figure 8. In general, these distributions are similar, with the most numerous weight category consisting of empty vehicles weighing up to 15,000 kg. The proportion of both fully loaded vehicles (40,000 kg) and overloaded vehicles (GVW > 40,000 kg) is highest in 2022. Data for 2023 and 2024 indicate a similar proportion of vehicles in both weight categories (fully loaded and overloaded).

**Figure 9:**
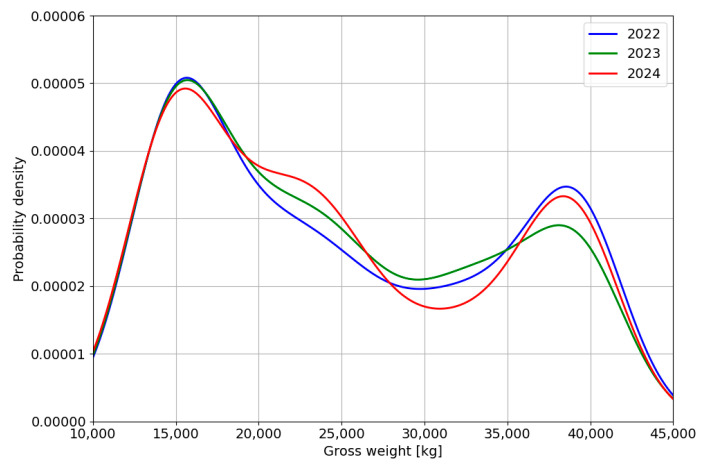
Gross vehicle weight spectra for vehicles in the reference category obtained from data recorded in each year in May.

For a detailed comparison of the results, considering the station’s location in southern Poland, data from the months of May 2022, May 2023, and May 2024 are used to assess changes in the systematic error of dynamic weighing at the station. This selection ensures the minimisation of the impact of temperature variability on the readings and allows for direct analysis of the results in relation to additional verification tests conducted during these months. The calculated gross vehicle weight spectra for the reference vehicle category, based on data collected in the respective years, are presented in Figure 9.

The analysis of the total weight distribution results leads to the identification of certain changes in the distributions for each of the analysed periods. The distribution for the 2023 data is characterised by a reduction in the second density peak and, at the same time, slightly higher values of the probability density function in the weight categories ranging from 20,000 to 35,000 kg compared to the distribution for the 2022 data. In contrast, the distribution obtained for the 2024 data shows a strong similarity in both density peaks when compared to the 2022 distribution. At the same time, the changes in the probability density function values for the weight categories between 20,000 and 28,000 kg are noticeably larger than those for the earlier years, while for the weight categories between 28,000 and 35,000 kg, they are clearly smaller than for the previous years. These changes in the distribution may be caused by changes in traffic characteristics (shifts in the proportion of vehicles in different weight categories) or potentially due to sensor drift and changes in systematic weighing error. Excluding these factors would require, as a first step, an analysis of the values and distribution of load.

### 3.3. The Steering Axle and Wheel Load Spectra for the Reference Vehicle Category

**Figure 10:**
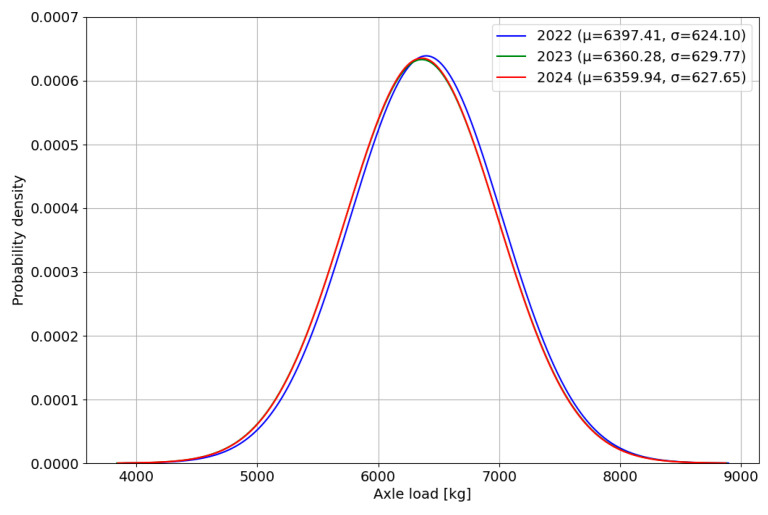
Steering axle load spectra for the reference vehicle category, obtained based on data recorded in each year, specifically in the month of May.

The calculated steering axle load spectra for the reference vehicle category are presented in Figure 10. It can be observed that the differences between the expected values for the given distribution are below 1% of the highest value in the analysed dataset obtained from data registered in 2022. The axle load distributions for 2023 and 2024 are nearly identical, showing comparable expected values and standard deviations. In the distribution for 2022, the expected value of axle load was slightly higher. This result leads to the hypothesis of high stability of the measurement system during the analysed period.

**Figure 11:**
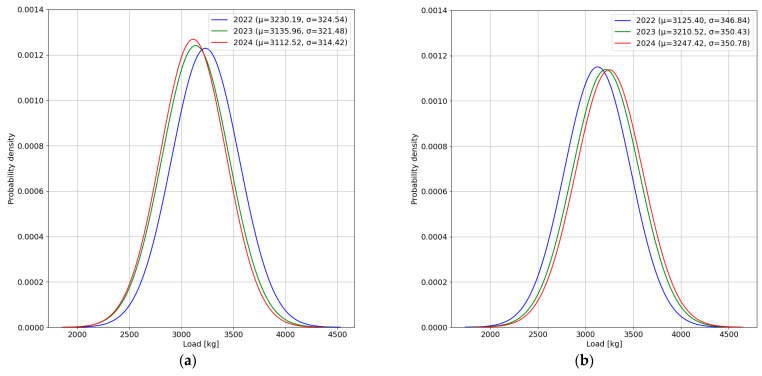
Wheel load spectra for the steering axle of the reference vehicle category, obtained based on data recorded in each year in May: (**a**) left wheel load; (**b**) right wheel load.

To confirm the hypothesis, information about the load of a single wheel on the first axle of the reference vehicle category was selected from the raw data. The calculated wheel load spectra for the steering axle of the reference vehicle category are presented in Figure 11.

The analysis of the distributions presented in Figure 11 indicates changes in the sensor performance over the analysed periods. The load distributions recorded for the left wheel are slightly more varied compared to the distributions recorded for the right wheel. In the case of the right wheel, the expected values of the wheel load distribution show an upward trend in the subsequent years. The difference between the expected values of the wheel load distribution in the considered periods exceeds 3.5% of the highest value obtained from the data recorded in 2024. The standard deviation of the load distribution remains nearly the same across the data from each year. On the other hand, for the left wheel, the expected values of the wheel load distribution show a downward trend in subsequent years. As a result, the expected value of the wheel load distribution in the analysed period of 2024 is 3.7% lower than the expected value obtained from the data recorded in 2022. The standard deviation of the load distribution for the data from 2022 and 2023 remains almost unchanged. The standard deviation of the load for the left wheel in the data recorded during the 2024 period is more than 3% smaller than the values determined for the earlier periods. In summary, over the years 2022–2024, systematic changes in the recorded values were observed for both the left and right sensors. At the same time, these changes compensate for each other, ensuring that the axle load is measured with practically unchanged accuracy.

### 3.4. The Results of Control Runs in 2023

**Figure 12:**
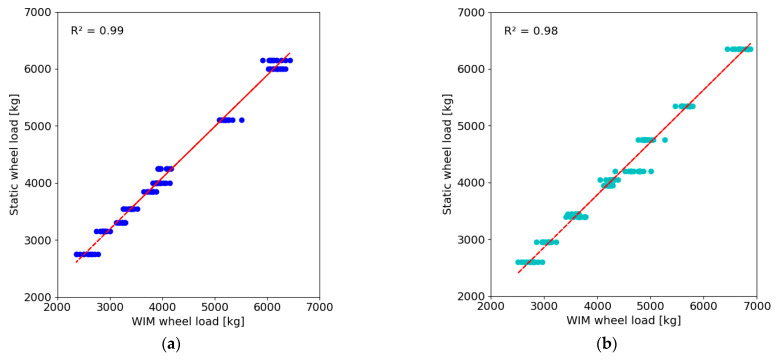
Comparison of wheel loads recorded in the 2023 in relation to the values determined on static scales: (**a**) left side; (**b**) right side. Dots represent measurement points, while the line represents the result of linear approximation.

The control tests conducted between the analysed periods in 2022 and 2023 indicate that the dynamic weighing error, in relation to the statically determined values, ranged from −0.3% to 3.9% for gross vehicle weight and from −4.8% to 5.9% for the first axle loads. Figure 12 presents a comparison of the recorded left and right wheel loads of the test vehicles in relation to the values determined on static scales.

**Figure 13:**
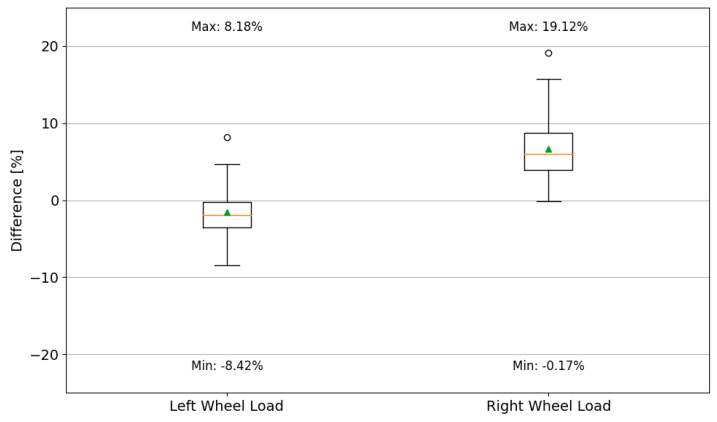
The percentage error in determining the wheel load for the reference category vehicle in the control tests of 2023 (box boundaries correspond to the IQR; whiskers—quartile 1 − 1.5 IQR and quartile 3 + 1.5 IQR, respectively; the dash inside the box represents the median, while the green triangle indicates the mean value).

The correlation coefficient for dynamically and statically determined wheel loads is comparable for the results obtained for both the left and right wheels. A comparison of the percentage error in determining the single-wheel load for vehicles of the reference category is shown in Figure 13.

Analysing the results presented in the form of a boxplot in Figure 13, it can be observed that there is a larger spread of measurement errors recorded for the wheels positioned on the right side of the vehicle. The median value of the error for the left side is −1.89%, with the box covering values from −3.5% to −0.2%. In contrast, the median error for the wheels on the right side of the vehicle is 6.04%, with the box covering values from 3.94% to 8.72%. The error for the total weight measured for the reference category vehicle ranged from 0.8% to 3.9%, while the error for the first axle load ranged from −3% to 1%.

### 3.5. The Results of Control Runs in 2024

**Figure 14:**
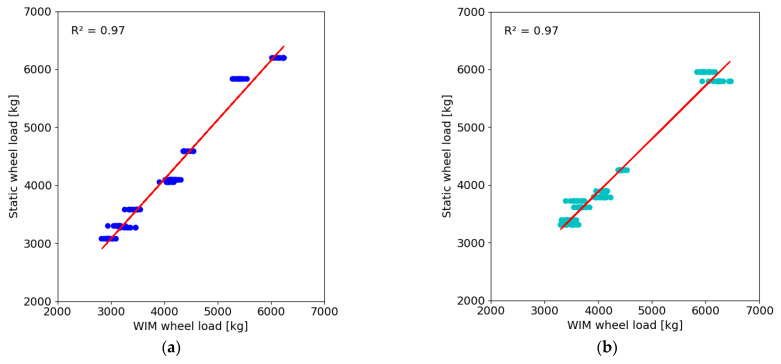
Comparison of wheel loads recorded in the 2024 in relation to the values determined on static scales: (**a**) left side; (**b**) right side. Dots represent measurement points, while the line represents the result of linear approximation.

Control tests conducted between the analysed periods in 2023 and 2024 indicate that the dynamic weighing error, in comparison to the statically determined value, ranged from −5.8% to 3.1% for the total weight and from −6.2% to 2.3% for the first axle loads. Figure 14 presents a comparison of the recorded left and right wheel loads of the test vehicles in relation to the values determined by static scales.

**Figure 15:**
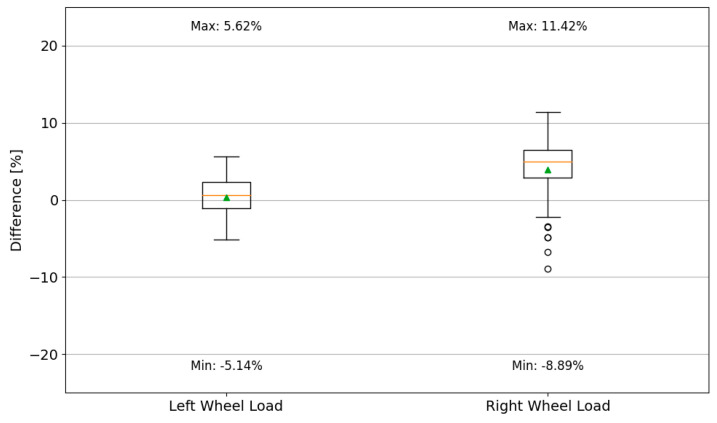
The percentage error in determining the wheel load for the reference category vehicle in the control tests of 2024 (box boundaries correspond to the IQR; whiskers—quartile 1 − 1.5 IQR and quartile 3 + 1.5 IQR, respectively; the dash inside the box represents the median, while the green triangle indicates the mean value).

The correlation coefficient between the dynamically and statically determined wheel loads is the same for both wheels. Compared to the results of the first control test, the correlation coefficients are slightly reduced. Figure 15 presents a comparison of the percentage error in the determination of single-wheel loads for the reference category vehicles.

Analysing the results presented in Figure 15, it is evident that there is a significantly increased spread of measurement errors recorded for the wheels positioned on the right side of the vehicle. The median error value for the right side is 4.94%, with the box encompassing values from 2.9% to 6.52%. In contrast, the median error for the left-side wheels is 0.61%, with the box encompassing values from −1.06% to 2.28%. The error for the total weight determined for the reference category vehicle ranged from 0.5% to 3.1%, while the error for the first axle load ranged from −6% to 1%.

## 4. Conclusions

The stability of load sensor operation in weigh-in-motion (WIM) systems plays a key role in the reliability of the entire measurement system and is particularly important in ensuring the expected accuracy during the long-term operation of the station. The use of indicators such as steering axle load spectra and gross vehicle weight spectra enables the assessment of variability in measurements across different operational periods of the station.

The analysis conducted for the examined station showed that the strain-gauge sensor system used from 2022 to 2024, despite the lack of calibration, allowed for measurements with the expected accuracy of class B(+7), meaning that the absolute error of the total weight did not exceed 7%. However, it was observed that changes in the surface geometry, including the formation of ruts exceeding the required level, affected the increased spread of results for the right wheel. Nonetheless, this has not led to the exceeding of the permissible error limit, and as a result, the measurement accuracy class on the individual sensor line has been maintained.

In the context of the expected functionality of WIM systems as direct enforcement systems, monitoring the stability and variability of load sensor readings is a crucial preventive action that enables control over the level of systematic weighing errors. The analysis of total weight distributions and axle loads for the reference vehicle category allows for flexible decision-making regarding the intervals at which stations should undergo calibration. In the case analysed in this study, the recommended calibration interval of 12 months is not justified. Its absence did not result in a loss of the expected station accuracy. As part of the next steps, we plan to consider a four-sensor in-line layout, which will enable us to consider the consistency of load values recorded by individual sensor lines in the evaluation of the system stability.

## Figures and Tables

**Table 1:** Average traffic volume at WIM station.

Parameter/Year	2022	2023	2024
Avg. traffic volume [veh./day]	7885	6938	6780
Vehicles with GVW > 3500 kg [%]	13	12	11

## Data Availability

The data presented in this study are available on request from the corresponding author. The data are not publicly available due to the size of the dataset.
